# A multimodal group-based immersive virtual reality intervention for improving cognition and mental health in patients with post-covid-19 condition. A quasi-experimental design study

**DOI:** 10.3389/fpsyg.2024.1441018

**Published:** 2024-07-25

**Authors:** Neus Cano, Josep Gómez-Hernández, Mar Ariza, Toni Mora, David Roche, Bruno Porras-Garcia, Maite Garolera

**Affiliations:** ^1^Brain, Cognition and Behavior Research Group, Consorci Sanitari de Terrassa (CST), Terrassa, Spain; ^2^BrainXRLab, Department of Psychology, Universitat Internacional de Catalunya, Sant Cugat, Spain; ^3^Unit of Medical Psychology, Department of Medicine, Universitat de Barcelona, Barcelona, Spain; ^4^Research Institute for Evaluation and Public Policy (IRAPP), Universitat Internacional de Catalunya, Barcelona, Spain; ^5^Neuropsychology Unit, Consorci Sanitari de Terrassa (CST), Terrassa, Spain

**Keywords:** immersive virtual reality, post-COVID-19 condition, cognitive training, physical activity, mindfulness

## Abstract

**Introduction:**

Adults with Post-COVID-19 Condition (PCC) may show cognitive impairments in attention, processing speed, memory, and executive function. Multimodal programs that combine cognitive training, physical activity and emotional tasks, such as mindfulness-based interventions (MBIs), may offer a suitable alternative for improving PCC treatments. Immersive Virtual Reality (IVR) is a promising technology that can enhance traditional cognitive training, physical activity, and MBIs. The use of IVR technology may increase engagement with these interventions and potentially enhance the individual benefits of cognitive training, exercise and MBIs. The current study evaluated the impact of a multimodal IVR intervention, comparing this with a usual care intervention (control group), in order to assess changes in cognition and mental health in adults with PCC. We also aimed to assess user experience factors such as enjoyment, perceived improvement, and fatigue following each multimodal IVR session within the experimental group.

**Method:**

Thirty-one participants with PCC symptoms were assigned to either the experimental group (IVR, *n* = 15) or the control group (usual care intervention, *n* = 16) in a quasi-experimental design study. The multimodal IVR intervention consisted of MBI, cognitive training and physical exercise and was delivered in a 60-min group session with 5 participants, twice a week, for 8 weeks (16 sessions in total). Measures of global cognition, attention, processing speed, verbal episodic memory and subjective memory complaints (primary measures), and depressive and anxiety symptoms and fatigue (secondary measures) were assessed at baseline and also after 8 weeks (post-intervention).

**Results:**

Mixed between-group (group) and within-group (pre-post assessments) ANOVAs revealed significant group*time interactions in global cognition, simple attention, processing speed, memory and depressive symptoms, with large effect sizes (*p* < 0.05; partial η^2^ > 0.14). There was also a marginally significant group*time interaction for executive function (*p* = 0.05). Follow-up analyses comparing pre-and post-intervention outcomes for each group separately showed that the experimental group significantly improved in global cognition, processing speed, memory and depressive symptoms, while the control group showed no significant pre-post changes. Friedman tests showed a significant main effect of time (χ^2^(2) = 6.609, *p* = 0.04), with a gradual increase in enjoyment from the first, to the mid, and then to the final session. In addition, perceived improvement scores remained high throughout the intervention, and patient-reported fatigue levels did not fluctuate significantly throughout the intervention.

**Conclusion:**

To our knowledge, no previous research has combined cognitive training, physical exercise and MBI using an IVR paradigm in adults with PCC. Despite their inherent limitations, our findings mark a pioneering step toward improving cognition and mental health outcomes in PCC through the innovative use of new technology and multimodal approaches. This first study should be accompanied by more extensive, randomized clinical trials aimed at further exploring and refining these interventions.

## 1 Introduction

The COVID-19 pandemic continues to pose enormous challenges, not only in terms of vaccination and infection prevention, but also in terms of the impact on the mental health and well-being of citizens and the persistence of symptoms in survivors (World Health Organization, 2022). Post-COVID-19 Condition (PCC) is an important issue for patients, physicians and society because it causes disability or reduces quality of life. It includes signs and symptoms that persist or occur beyond 12 weeks after severe acute respiratory syndrome coronavirus-2 (SARS-CoV-2) infection and cannot be attributed to alternative diagnoses (Shah et al., 2021). PCC can occur after severe, mild or even asymptomatic SARS-CoV-2 infection (Gupta et al., 2020). Possible causes of persistent symptoms include initial organ damage, persistent inflammation, viral activity, and ineffective antibody responses (Carda et al., 2020). Other factors may include poor fitness, pre-existing health conditions, mental disorders and negative lifestyle changes induced by the pandemic (Galea et al., 2020).

Available data on the incidence and course of PCC are heterogeneous due to differences in populations and the research approaches used. It has, however, been estimated that about 10–15% of adults (Davis et al., 2021) experience persistent symptoms (e.g., fatigue, dyspnea, anosmia or ageusia, brain fog, impaired cognition, sleep disturbances, anxiety or depression) and worsening quality of life for weeks or months after initial infection with SARS-CoV-2 (Mazza et al., 2020; May, 2022). A study of 3,762 respondents in 56 countries, conducted 7 months after COVID-19 infection, found that 85% of participants with PCC experienced “brain fog” and cognitive dysfunction (Mazza et al., 2020). Objective neuropsychological assessments of people with PCC have also shown impairments affecting attention, processing speed, memory, and executive function (Ariza et al., 2022; Delgado-Alonso et al., 2022; May, 2022). The brain fog and associated cognitive dysfunction described by patients share similarities with post-traumatic brain injury (Merritt et al., 2015) and the brain fog associated with chemotherapy (Cascella et al., 2018) and chronic fatigue syndrome (Ocon, 2013).

The World Health Organization has called on countries to prioritize rehabilitation of the cognitive, physical and emotional consequences of COVID-19 in the medium and long term (World Health Organization, 2022). Multimodal programs that combine cognitive training, physical activity, and emotional tasks, such as mindfulness, may offer a suitable alternative for improving PCC treatment. Cognitive training has been shown to improve specific cognitive functions, whether this focuses on working and episodic memory, executive function, or information processing speed (Ball et al., 2002; Brehmer et al., 2012). These approaches have been shown to be effective in attenuating cognitive decline and improving cognitive function in older adults (Zelinski et al., 2011), and in cases of mild cognitive impairment and dementia (Barnes et al., 2009), acquired brain injury (Hooft et al., 2005), chronic fatigue syndrome (Maroti et al., 2015), and post-COVID syndrome (García-Molina et al., 2021). Cognitive training interventions are often combined with physical activity to improve cognitive function (Zhu et al., 2016). The implementation of personalized and supervised exercise programs is considered a viable, comprehensive intervention and one that can be tailored to different PCC cases and symptoms (Cattadori et al., 2022; Schaefer and Khanna, 2023). This approach may not only alleviate the intensity of the immediate infection but also help to reduce long-term symptoms after COVID-19 infection (Barbara et al., 2022; Jimeno-Almazán et al., 2022; Romanet et al., 2023). Finally, mindfulness is defined as paying attention in a particular way: intentionally, in the present moment, and nonjudgmentally (Kabat-Zinn, 2005). Mindfulness-based interventions (MBIs) have been shown to improve attention, working memory, verbal fluency and response inhibition in healthy adults (Chiesa et al., 2011), to enhance well-being and emotion regulation (Grossman et al., 2004), and to improve anxiety and depressive symptoms (Hofmann et al., 2010). Recent studies have also investigated the potential of MBIs for PCC, highlighting their usefulness in addressing the emotional and cognitive challenges associated with the condition (Porter and Jason, 2022; Hausswirth et al., 2023).

Immersive virtual reality (IVR) is a promising technology that can enhance traditional cognitive training, physical activity and MBIs. It can be applied with varying degrees of immersion. IVR typically provides a computer-generated 360° virtual world (i.e., a full field of view) through an immersive display device (Slater and Wilbur, 1997). Several studies have focused on the utility and efficacy of IVR cognitive training interventions in vulnerable populations. A recent systematic review and meta-analysis reported that the use of virtual reality (VR) exergames (games that involve physical movement) with older adults resulted in significant gains in their cognitive function and memory, as well as improvements in their depressive symptomatology (Yen and Chiu, 2021). These findings support other meta-analyses that showed similar results with exergames (Li et al., 2016; Cugusi et al., 2021). Other systematic reviews have shown that IVR cognitive training can be an important resource in the treatment of neurocognitive disorders. Patients report significant gains in cognition (e.g., memory and dual-tasking) and psychological well-being (Moreno et al., 2019; Riva et al., 2020). Another systematic review highlighted similar findings in individuals at risk of cognitive decline (Coyle et al., 2015).

IVR technology has also begun to be applied to patients diagnosed with COVID-19. A recent literature review showed that IVR games can potentially improve functional and cognitive outcomes, increase satisfaction among COVID-19 patients, and empower patients to take greater control of their healthcare (Ahmadi Marzaleh et al., 2022). Consistent with these findings, another study found that using a multimodal IVR system to provide guided meditation, exploration of natural environments, and cognitive stimulation games offered a viable approach for both patients and healthcare providers, as participants acknowledged that this had the potential to help them to better cope with isolation and loneliness (Kolbe et al., 2021). In addition, a recent study of a 6-week IVR home-based exercise intervention, in which participants were able to choose to engage in either VR-based relaxation or cognitively challenging exercises, revealed that home-based VR exercise was feasible, safe and well-accepted by a significant proportion of patients with COVID-19 (Groenveld et al., 2022).

To our knowledge, there have been no previous studies that have included cognitive training, physical exercise, and MBI conducted with adults with PCC. If these different interventions can have positive cognitive and neural effects when used individually, then combining them could be expected to produce summative effects. In addition, the use of IVR technology may increase engagement with these interventions and provide a more immersive and interactive environment, potentially enhancing the individual benefits of cognitive training, exercise and MBIs.

The primary objective of this pilot study was to present a groundbreaking multimodal intervention that combines cognitive training, physical exercise and MBIs, using MK360 IVR technology. This is the first implementation of a group-based IVR intervention for adults with PCC. The current study aims to evaluate its effects by comparing a multimodal IVR intervention (i.e., cognitive training, physical exercise and MBI) with a usual care intervention (referred to as a control group) in order to assess changes in cognition and mental health in adults with PCC. We hypothesized that participants who receive the multimodal IVR intervention would show post-intervention improvements in cognitive domains (e.g., global cognition, attention, processing speed, memory, executive function) and secondary measures (e.g., anxiety and depressive symptoms, and fatigue) compared with those in the control group.

In addition to our primary objective, we also aimed to assess user experience factors such as enjoyment, perceived improvement and fatigue following each multimodal IVR session conducted within the experimental group. We hypothesized that participants would report progressively higher levels of enjoyment, perceived improvement and reduced fatigue over the course of the intervention sessions. In addition, if we found improvements in cognitive domains (e.g., global cognition, attention, processing speed, memory, executive function) and mental health (e.g., anxiety and depressive symptoms), we then assessed whether these improvements were associated with a greater generalization of the results to daily life situations and activities.

## 2 Methods

### 2.1 Study design

The study was a longitudinal, parallel, open-label, non-randomized, pilot study of adults diagnosed with PCC. Participants were sequentially assigned to two groups. A total of 15 participants were assigned to the experimental group (IVR multimodal intervention) and 16 to a control group (usual care intervention). Enrollment was conducted for 5 months, between March 2022 and August 2022.

### 2.2 Participants

A total of 31 adults with PCC, from eight public primary care centers belonging to the Consorci Sanitari de Terrassa (Terrassa Health Consortium, Barcelona, Spain), participated in the study (76.92% women, 23.08% men; *M*_age_ = 50.31 years, *SD* = 6.29 years; *M*_onset_Covid19_ = 14.60 months, *SD* = 7.67 months). All the patients enrolled in the study were over 18 years of age and met the criteria for PCC. All the participants presented symptoms which included fatigue, difficulty thinking or concentrating (i.e., brain fog), palpitations, muscle and/or joint pain, respiratory problems, pins and needles, bowel dysfunction, insomnia, loss of smell and/or taste, hair loss, and rash (Nalbandian et al., 2021). The participants also had to report any incidences of anxiety or depressive symptoms, as assessed by the Patient Health Questionnaire-9 (PHQ-9) and Generalized Anxiety Disorder-7 (GAD-7) scales; only participants who scored ≥6 on the PHQ-9 and/or ≥ 10 on the GAD-7 were included in the study. Finally, the patients had to be able to understand Spanish or Catalan and had to give their informed consent to participate in the study.

Exclusion criteria for this sample were pre-existing psychiatric, neurological, neurodevelopmental or systemic disorders that caused cognitive deficits, and also motor or sensory impairments that might have prevented them from completing the program (e.g., pronounced dysarthria, paresis, problems in the visual and/or auditory fields). The participants did not receive any compensation for participating in the study, and were all covered by research insurance.

### 2.3 Measures

All the primary and secondary measures were assessed by two un-blinded clinical neuropsychologists, who were supervised by a senior neuropsychologist. The demographics collected included age, sex and years of education. The Word Accentuation Test (WAT) was used to estimate the premorbid intelligence quotient (Gomar et al., 2011), following the procedure adopted in previous studies conducted with similar populations (Ariza et al., 2023).

The following primary measures were considered:

- *Subjective memory complaints* were assessed using the Memory Failures of Everyday (MFE) a 28-item self-report questionnaire with a score ranging from 0 to 84, with higher scores indicating more frequent memory failures (Montejo et al., 2014).
- *Global cognition* was assessed using the Spanish version of the Montreal Cognitive Assessment (MoCA), a screening tool designed to identify mild cognitive impairment and other cognitive deficits (Ojeda et al., 2016).
- *Attention:* Inattention, impulsivity, sustained attention and vigilance were assessed using the computerized (second) version of the Continuous Performance Test (CPT-II; Conners and Sitarenios, 2011), in which a series of visual stimuli are presented and the participant is asked to respond when a specific stimulus appears. Three main outcomes were considered: CPT-II omissions, which represent missed target stimuli; CPT-II commissions, which represent incorrect responses to non-targets; and CPT-II reaction time, which reflects the speed of correct responses.
- *Auditory attentional capacity* was assessed using the Wechsler Adult Intelligence Scale, 4th edition (WAIS-IV) Digit Span Forward test (Wechsler, 2008), in which participants are presented with a series of digits and asked to repeat the digits in the order in which they were presented. *Selective attention* was measured using the Trail Making Test - Part A (Reitan, 1958), in which participants were asked to connect numbers in sequential order as quickly as they could.
- *Processing speed* was measured using the WAIS-III Digit Symbol Coding subtest (Wechsler, 1991). In this task, participants are given a key that pairs numbers (typically 1–9) with specific symbols and are asked to fill in the corresponding symbols for a series of numbers as quickly and accurately as possible, within 120 s. The Stroop Words and Stroop Colors subtests (Llinàs-Reglà et al., 2013) were also used to assess processing speed. These consist of reading color names printed in black ink (Words) and identifying the color of the ink used to form various colored rectangles (Colors).
- *Verbal episodic memory* was assessed using the Rey Auditory Verbal Learning Test (RAVLT; Bean, 2011; Alviarez-Schulze et al., 2022). The test involves the presentation of two lists of words (A and B) and the participant is asked to recall as many words as possible after each presentation.
- The main subdomains relating to different *executive functions* were also assessed. *Working memory* was assessed using the Digit Span Test Backward, a subtest of the WAIS-IV, in which participants are given a series of digits and asked to repeat them backward (51). *Verbal fluency* was assessed using the Controlled Oral Word Association Test (COWAT; Peña-Casanova et al., 2009) and the ANIMALS category of the Semantic Verbal Fluency test (Ardila et al., 2006), which assess the ability to generate as many words as possible (e.g., words beginning with F, A, and/or S, or the names of animals) within an established time frame. *Response inhibition, cognitive flexibility and set-shifting* ability were assessed by the Trail-Making Test - part B (Reitan, 1958), which requires participants to rapidly connect alternating numbers and letters in sequential order. The Stroop Color-Word Test (Llinàs-Reglà et al., 2013) was also used, which requires participants to identify the color of a printed word while ignoring the meaning of the word itself, which typically denotes a different color.

The following secondary measures of mental health and well-being were also considered:

- *Mental health* status was assessed using validated Spanish versions of the PHQ-9 (Diez-Quevedo et al., 2001; Kroenke et al., 2001) and GAD-7 (Spitzer et al., 2006; García-Campayo et al., 2010) scales, which assess the severity of depressive and anxiety symptoms identified in the 2 weeks prior to the test. The PHQ-9 consists of nine items, with each item rated on a scale from 0 (not at all) to 3 (almost every day), and having a total score ranging from 0 to 27, with higher scores indicating more severe depression. The GAD-7 is a self-report measure consisting of 7 questions based on the frequency of the detection of symptoms of anxiety over the 2 weeks prior to the test. The total score ranges from 0 to 21, with higher scores indicating more severe symptoms of anxiety.
- *Fatigue* was assessed using the Chalder Fatigue Scale (CFQ 11; Jackson, 2015), which consists of 11 items divided into two subscales: physical fatigue (7 items) and mental fatigue (4 items). Respondents rate each item based on their experience over the previous month, using a 4-point Likert scale, resulting in a total score ranging from 0 to 33. Higher scores indicate greater fatigue severity.

To assess the user experience and the generalizability of our findings, we designed two custom-made assessment tools.

- *User Experience*: We developed a 9-item user experience scale which was to be completed after each IVR session. The scale uses a 5-point rating system, ranging from 1 (not at all) to 5 (a lot), and we calculated scores for three primary domains: user enjoyment, perceived improvement and rest-fatigue, with each ranging from 3 to 15. The user enjoyment domain includes aspects such as satisfaction, enjoyment and happiness during sessions, with higher scores indicating greater enjoyment. The perceived improvement domain includes cognitive, emotional and physical improvements, again with higher scores indicating a better user experience. Finally, the rest fatigue domain includes cognitive, emotional and physical exhaustion experienced during the session, with higher scores indicating less fatigue. Although the user experience was assessed in each session, for the purposes of this study, we only considered measurements taken during the first, third and final sessions.
- *Generalizability of Results*: We developed a comprehensive 32-item scale to assess the transferability of cognitive improvements to everyday situations and tasks, addressing a variety of specific PCC cognitive challenges and experiences, such as mental fog, forgetfulness, impulsivity, distractibility, learning difficulties, problem solving, multitasking and self-initiation in real-life contexts. The scale uses a 5-point rating system, with scores ranging from 1 to 5, and we calculated a total score by aggregating the individual item scores, resulting in a minimum score of 32 and a maximum score of 160.

### 2.4 Procedure

**Figure 1:**
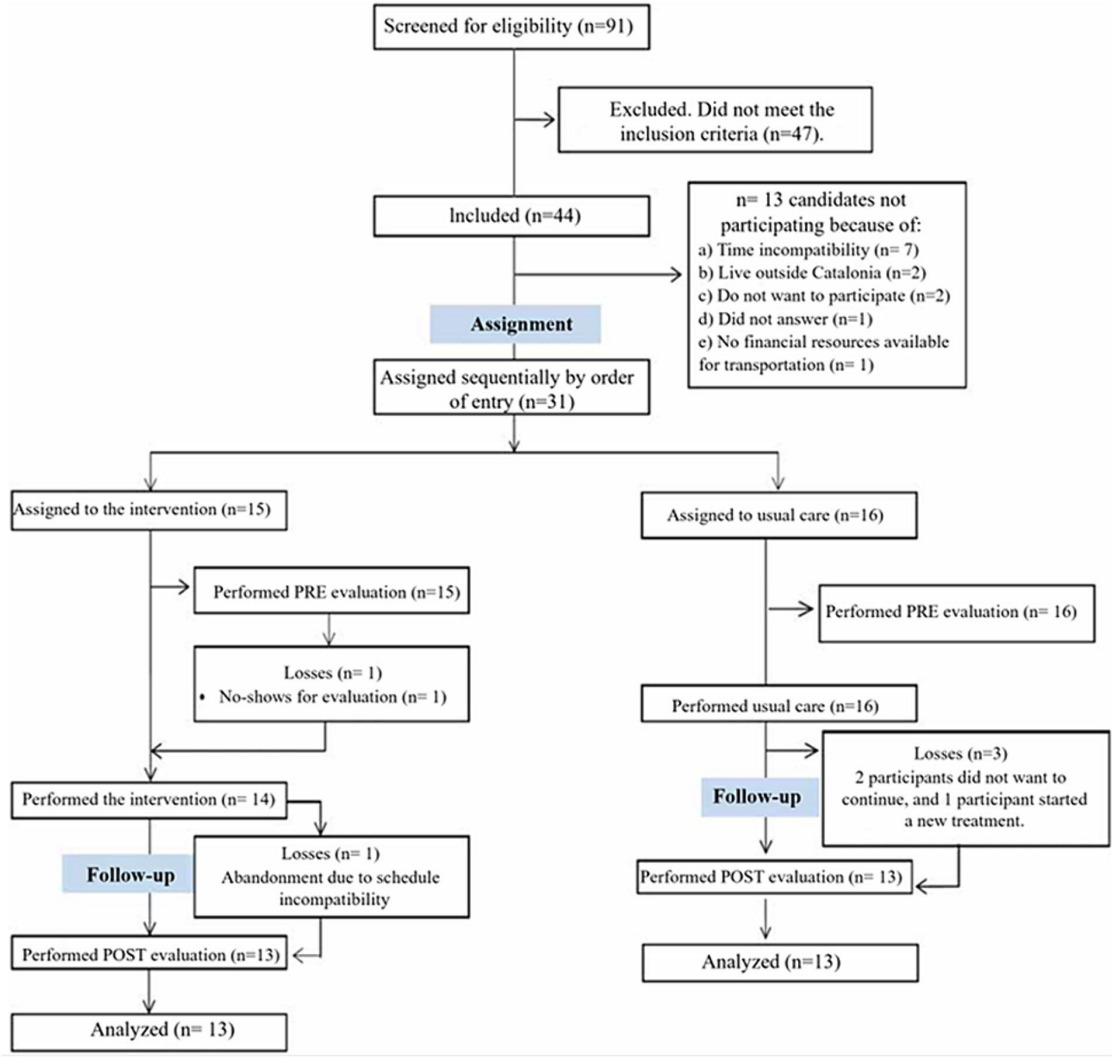
Participant workflow throughout the pilot study.

Following a comprehensive explanation of the study to potential adult participants (i.e., individuals diagnosed with PCC), an initial eligibility screening visit was conducted by a clinical neuropsychologist to further evaluate other inclusion and exclusion criteria. Eligibility screening included a clinical interview to assess the progression of PCC symptoms. Of the 91 participants contacted, only 44 met the eligibility criteria. Of these, only 31 were ultimately interested in participating in the study and provided informed consent. As shown in Figure 1, patient participation in the study was monitored at baseline and then throughout the study until completion of the post-clinical evaluation.

Study completion was defined as participants attending 80% or more of the scheduled sessions. Participants in the experimental group who did not reach this threshold, specifically one individual (*n* = 1), were excluded from the study. All primary and secondary measures were collected at two different time points: first, at baseline (pre-clinical assessment), and then after 8 weeks (post-clinical assessment), for both the control and experimental groups. In addition, participants in the experimental group completed the User Experience Assessment after each IVR session and the Generalizability of Results Test after assessing the impact of the intervention on the generalization of acquired skills relating to real-world situations.

The multimodal IVR intervention lasted 8 weeks and took place twice per week, for 60 min (with 16 sessions per participant). The intervention was delivered using MK360 IVR technology. The MK360 is an immersive projection system developed by the company Broomx Technologies and it has been used to transform a room into a multi-sensory, interactive space. This immersive experience can be shared among several people without using headsets, and it allows users to enjoy 360°videos and interactive virtual reality applications in physical spaces, socially and headset free. Its state-of-the-art optics display images over 3 walls and the ceiling of a wide range of indoor spaces. The devices include a projection module, CPU, GPU, integrated speaker, WiFi hotspot and various connection options. The MK360 devices are compact, portable and easy to install and operate on a mobile, tablet or computer with the Broomx software and content platform.

**Figure 2:**
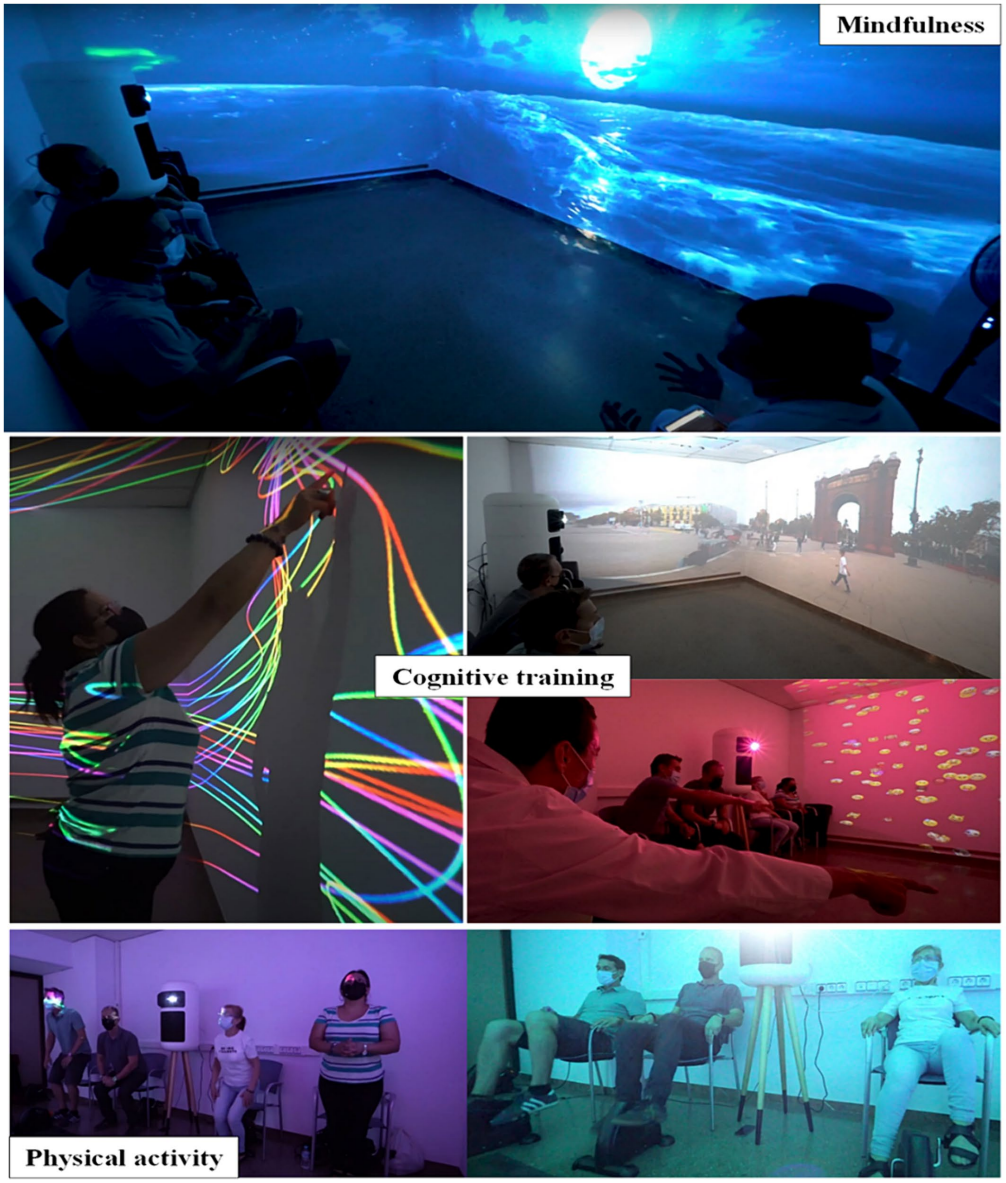
Visual examples of mindfulness, cognitive and physical tasks, conducted within the IVR sessions.

The intervention was conducted in a group session involving 5 participants and was supervised by an expert in neuropsychology Each 60-min program session included three activities: mindfulness, cognitive training, and physical exercise (see Figure 2). Each of the 16 program sessions was unique, but the order and duration of the blocks were similar.

During the first 10 min of each session, the neuropsychologist explained the purpose of the session and the process involved. This explanation included the cognitive and emotional aspects affected by the existing pathology (if present) and the importance of applying the knowledge and practice gained in the sessions to daily life. Participants were also informed of the importance of reflecting on their thought processes and how they learn, so that they would become more aware of their learning strategies and understand the reasons behind the positive or negative outcomes of a particular activity.

- Mindfulness (10 min): An adaptation of the Mindfulness-Based Stress Reduction program developed by Jon Kabat-Zinn at the University of Massachusetts (Kabat-Zinn, 1998; Bunjak et al., 2022) was used; it included body scanning, seated meditation and gentle Hatha yoga, with an emphasis on body awareness. In addition, the researchers explained that the purpose of the MBI was to help participants achieve a state of baseline functioning. This meant training them to maintain a certain level of activity and independence, regardless of any challenging circumstances they might face, such as difficulties related to their pathology or personal situation.
- Cognitive training (30 min): The cognitive training program used virtual environments that simulated real-life situations, such as walking in parks or tourist areas of the city, to improve cognition. The program worked sequentially on different cognitive areas such as attention, processing speed, episodic memory and executive function, increasing the difficulty of the exercises based on user performance. Three unique cognitive stimulation activities were used to specifically target these functions. The “Parc Güell” simulation promoted attention, social cognition and memory, by having participants report observed stimuli and recall details from the video. The “Crazy Lines” activity was designed to engage attention and improve processing speed and working memory by having participants follow numerical sequences related to color-coded lines. Finally, the “Emoticons” activity was designed to improve visual tracking, attention, processing speed and mental arithmetic, by asking participants to count or locate specific emoticons randomly placed around a room.
- Physical exercise (20 min): All participants completed a multimodal limb training and therapeutic exercise program that included balance work, stretching and progressive muscle strengthening exercises. The physical activation activities consisted of continuous repetitions: getting up from a chair and repeatedly sitting down (squats), pedaling (pedaling a bike), and climbing steps (up and down).

Usual Care Intervention: The control group continued receiving routine control visits and remotely administered psychoeducation guidelines to monitor their physical exercise and compliance with pharmacological treatments.

### 2.5 Data analyses

The following statistical analyses were performed using IBM SPSS Statistics 27.0 (IBM Corporation, Armonk, NY, United States). Descriptive statistics were computed for all the study variables. Initially, Fisher’s exact test for categorical variables, the chi-square test of homogeneity for polytomous ordered variables, and independent-samples t-tests for continuous variables, along with their nonparametric equivalents, were used to determine any significant differences between groups of different demographic and clinical variables at baseline/pre-assessment. Mixed between-group (group) and within-group (pre-post assessments) ANOVAs with Bonferroni corrections were then performed for primary and secondary measures. One-way *post hoc* analyses with Tukey corrections were used to assess differences between the multimodal IVR intervention and the pre-and post-intervention control conditions. The critical level of statistical significance was set at α = 0.05. Effect size was calculated using partial eta squared (partial η^2^). Graphs and descriptive statistics were used to examine assumptions. Homogeneity of variance was satisfied for most of the variables, as shown by Levene’s test. Although some of the variables were not normally distributed (e.g., TMT-B pre-assessment, RAVLT delayed recall pre-assessment, CPT omissions pre-and-post assessments, CFE pre-and-post assessment, MFE-Q pre-and-post assessments, CPT commissions post-assessment and Stroop words and colors subtest post-assessment), as determined by the Shapiro–Wilk test, it was decided to perform the analysis anyway as two-way mixed ANOVAs are considered to be reasonably robust tests for deviations from normality (Schmider et al., 2010).

In addition, we performed Friedman’s analysis, a nonparametric alternative to one-way repeated measures ANOVA, to examine changes in user experience scores reported by participants in the experimental group at three different time points: after the first, third and final sessions of the multimodal IVR intervention.

## 3 Results

Fisher’s exact test, the chi-square test of homogeneity and the independent-samples t-test showed that there were no significant differences between the groups at baseline for any of the demographic variables (Table 1). Independent samples t-test analyses showed statistically significant group differences (*p* < 0.05) in the pre-test session on episodic memory measures. The experimental group reported significantly greater memory dysfunctions in the RAVLT Sum (*t*(24) = 4,858, *p* < 0.001), RAVLT immediate recall (*t*(24) = 2,100, *p* = 0.046) and RAVLT delayed recall (*t*(24) = 2.727, *p* = 0.01) than in the control group. The mean and standard deviations of primary and secondary measures of the study are reported in Table 2.

**Table 1:** Descriptive results, including the demographic and clinical characteristics of the two groups.

	Variable	*EG* (*n* = *13*)	*CG* (*n* = *13*)		
		*M (SD)*	*M (SD)*	*t-test (df)*	*p*
Age (years)		49.85 (*2.09*)	50.77 *(5.01)*	*0.368 (24)*	0.72
Education (years)		11.77 (*3.11*)	13.85 *(3.62)*	*1.567 (24)*	0.13
Body mass index (kg/m^2^)		27.37 (7.60)	28.46 (5.88)	*0.401 (23)*	0.69
Onset Covid-19 (months)		13.08 (5.12)	16.00 (9.44)	*0.970 (23)*	0.34
IQ – WAT (Total score)^a^		23.25 (4.41)	24.15 (3.02)	0.602	0.53
		*n (%)*	*n (%)*	*X^2^ (df)*	*p*
Sex, n (%)				*N/A*	.64^b^
	Women	9 (69.23)	11 (84.61)		
	Men	4 (30.77)	2 (15.39)		
Ethnic, n (%)				*N/A*	.43^b^
	White-Caucasian	9 (69.20)	6 (46.20)		
	Hispanic-Latinx	4 (30.80)	7 (53.80)		
Severity COVID-19, n (%)				*N/A*	.64^b^
	Mild. Ambulatory care	9 (69.20)	11 (84.60)		
	Severe. Hospitalization / Intensive Care Units	4 (30.80)	2 (15.40)		
Vaccination status, n				*1.963 (2)*	0.37
	Non-vaccinated	2	1		
	1 dose	4	2		
	2 or 3 doses	6	10		
Number of comorbid health disorders^c^, n				*1.105 (2)*	0.76
	0 comorbid disorders	4	3		
	1 or 2 comorbid disorders	4	4		
	>2 comorbid disorders	4	6		
Medications^c^, n				*N/A*	*N/A*
	Cardiovascular/Metabolic Health	7	4		
	Mental Health/Neurological Disorders	4	4		
	Pain/Inflammation Management	2	2		
	Respiratory/Allergic Response	3	1		
	Other Conditions	3	7		
	None	2	1		

EG, Experimental; CG, Control group. M, Mean; SD, Standard Deviation. N/A, not applicable. ^a^IQ-WAT, Intelligence Quotient-Word Accentuation Test. ^b^Fisher’s exact test, ^c^Comorbid health disorders may include: Cardiopathy, Chronic renal insufficiency, Respiratory illness, Hypertension, Dyslipidemia, Diabetes Mellitus, Obesity, Thyroid affection, Hepatic illness, Systemic illness, HIV, Chronic pain, or other disorders.

**Table 2:** Mean and standard deviation of primary and secondary measures in the study.

	EG (*n* = 13)		CG (*n* = 13)	
	Baseline *M (SD)*	Post-assessment *M (SD)*	Baseline *M (SD)*	Post-assessment *M (SD)*
MoCA^a^	26.15 (2.91)	28.54 (1.45)	27.08 (1.89)	26.92 (1.85)
FAS^b^	42.77 (11.47)	48.54 (14.12)	45.15 (12.26)	48.00 (8.95)
ANIMALS^c^	9.08 (3.86)	9.00 (2.65)	9.92 (2.96)	10.46 (1.94)
DS-F^b^	43.62 (10.10)	47.38 (8.02)	44.77 (8.21)	42.85 (5.49)
DS-B^b^	48.69 (7.95)	51.46 (9.81)	48.23 (10.03)	44.23 (9.13)
TMT-A^c^	9.23 (4.76)	10.92 (4.09)	10.00 (2.16)	10.08 (2.33)
TMT-B^c^	8.92 (3.45)	10.54 (3.67)	8.85 (2.23)	9.08 (2.53)
Stroop-W^c^	8.08 (3.95)	8.38 (3.35)	8.62 (4.29)	8.69 (3.07)
Stroop-C^c^	7.54 (4.45)	10.00 (4.67)	10.23 (2.68)	8.85 (2.58)
Stroop W-C^c^	8.08 (3.63)	9.46 (3.99)	10.00 (1.87)	9.00 (1.96)
CPT-II O^b^	71.62 (50.91)	53.70 (16.63)	71.74 (56.46)	56.28 (18.64)
CPT-II C^b^	52.61 (13.18)	48.78 (14.19)	56.61 (15.22)	50.72 (13.74)
CPT-II RT^b^	55.87 (12.79)	59.00 (12.61)	55.65 (14.92)	54.63 (12.04)
Digit Symbol-Cod^c^	11.00 (3.39)	12.00 (2.94)	11.69 (1.97)	12.54 (1.90)
RAVLT-Sum^b^	37.31 (5.80)	48.38 (13.02)	55.45 (10.57)	48.00 (12.87)
RAVLT-IR^b^	41.46 (9.37)	47.85 (11.77)	50.73 (12.64)	47.36 (9.93)
RAVLT-DR^b^	41.62 (8.75)	48.92 (11.79)	53.09 (11.74)	47.91 (13.28)
MFE-Q	22.00 (15.53)	20.58 (11.73)	25.50 (16.58)	19.08 (11.40)
PHQ-9	14.38 (4.96)	8.38 (6.59)	9.25 (6.35)	9.17 (6.87)
GAD-7	9.08 (5.85)	6.23 (4.32)	8.00 (5.10)	7.83 (6.07)
CFQ-11	10.46 (1.66)	6.08 (4.50)	9.50 (2.84)	7.08 (3.42)

^a^raw direct scores, ^b^standardized T scores, ^c^scaled scores. MoCA, Montreal Cognitive Assessment; DS-F, Digit Span Forward; DS-B, Digit Span Backward; TMT, Trail-Making Test; A-B subtests; Stroop-W, Stroop Words; Stroop-C, Stroop-Colors; Stroop-WC, Stroop-Words and Colors; CPT-II, Continuous Performance Test II; O, Omissions; C, Commission; RT, Reaction Time; Cod, Digit Symbol Coding; IR, RAVLT Sum, Immediate Recall; DR, Delayed Recall; MFE-Q, Memory Failures of Everyday Questionnaire; PHQ-9, Patient Health Questionnaire-9; GAD-7, the Generalized Anxiety Disorder-7; CFQ, Chalder Fatigue Scale.

### 3.1 Cognition, mental health, and wellbeing

Mixed ANOVA analyses showed statistically significant interactions between group and assessment time in global cognition (MoCA; *F*(1, 24) = 5.500, *p* = 0.03, partial *η^2^* = 0.186), processing speed (Stroop colors; *F*(1, 24) = 7.895, *p* = 0.01, partial *η^2^* = 248), and episodic memory (RAVLT Sum; *F*(1, 22) = 11.491, *p* = 0.003, partial *η^2^* = 343), RAVLT immediate recall (*F*(1, 22) = 4.744, *p* = 0.04, partial *η^2^* = 0.177) and RAVLT delayed recall (*F*(1, 22) = 11.074, *p* = 0.003, partial *η^2^* = 335), with large effect sizes (*η^2^* > 0.14) (67). Some selective attention measures, such as the TMT-A, reached marginally statistically significant group*time interactions: (*F*(1, 24) = 3.926, *p* = 0.059, partial *η^2^* = 0.141) and executive function measures, such as the Stroop Colors and Words (*F*(1, 24) = 4.243, *p* = 0.05, partial *η^2^* = 0.150), but there was still a large effect size (Cohen, 1977).

**Figure 3:**
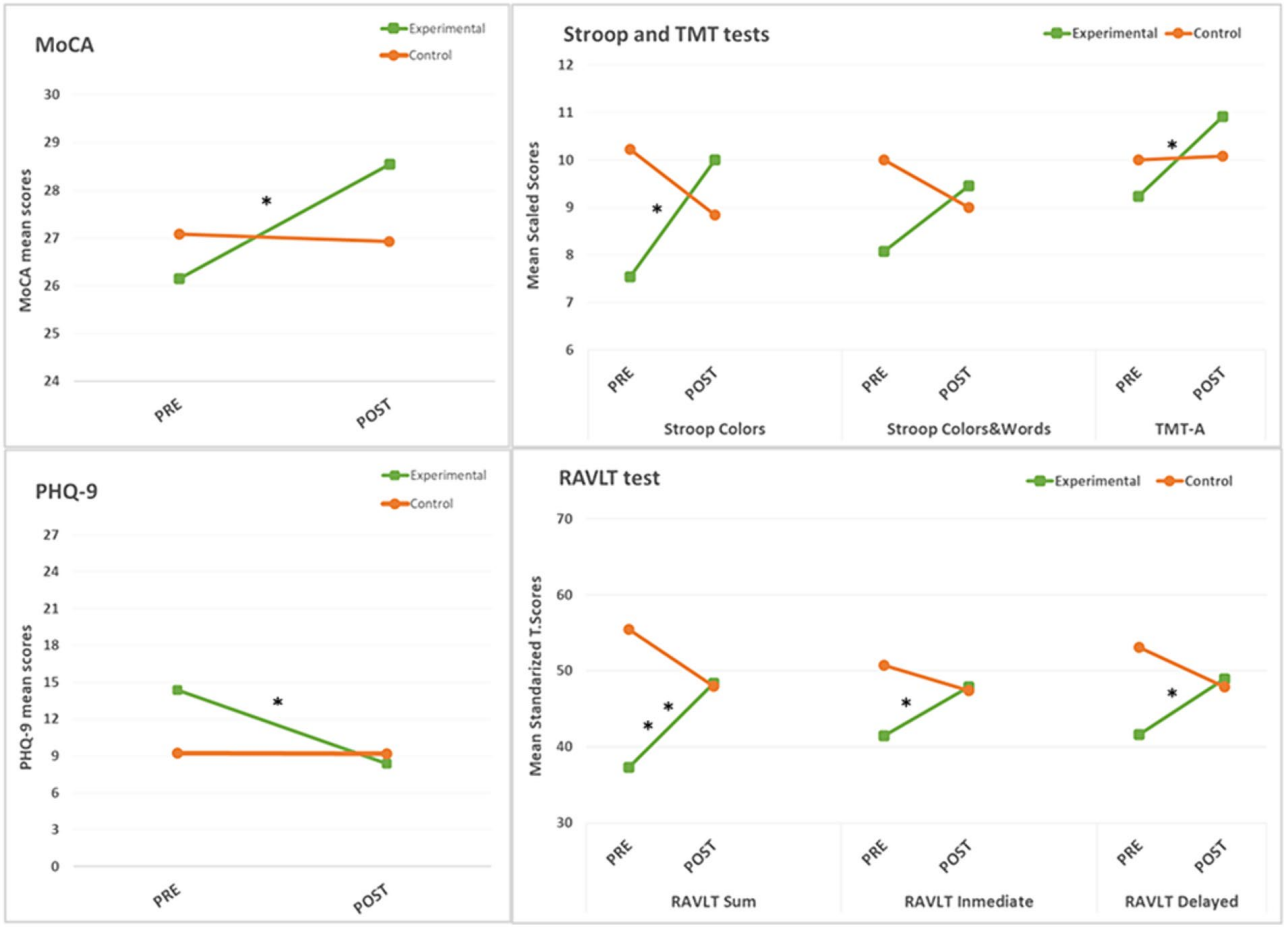
Mean group differences at baseline (PRE) and after the intervention (POST), in measures with significant mixed ANOVA group*time interactions. Asterisks denote significant pre-post changes (* = *p* < 0.05, ** = *p* < 0.01).

Follow-up analyses, with the mean differences (*MD*) and standard error (SE) reported, separately compared outcomes before and after the intervention for each group, and showed that the experimental group showed significantly improved scores in the MoCA (*MD* = −2.38, *SE* = 0.76, *p* = 0.005), Stroop Colors (*MD* = −2.46, *SE* = 0.96, *p* = 0.018), TMT-A (*MD* = −1.69, *SE* = 0.576, *p* = 0.007) and RAVLT Sum (*MD* = −11.07, *SE* = 3.701, *p* = 0.007), RAVLT immediate recall (*MD* = −6.38, *SE* = 3.03, *p* = 0.047), and RAVLT delayed recall (*MD* = −7.308, *SE* = 2.54, *p* = 0.009). In comparison, the control group showed no significant changes before and after the intervention in any of the cognition measures (Figure 3).

Mixed ANOVA analyses showed a statistically significant interaction between group and assessment time with regard to depressive symptoms, assessed by the PHQ-9 (*F*(1, 23) = 5,975, *p* = 0.03, partial *η^2^* = 0.206), with a large effect size (67). Follow-up analyses of the PHQ-9 showed a statistically significant improvement (*MD* = −6.00, *SE* = 1.66, *p* = 0.002) in the experimental group from baseline to post-assessment. In comparison, the control group reported no significant changes in depressive symptoms before and after the intervention (Figure 3).

### 3.2 User experience

**Figure 4:**
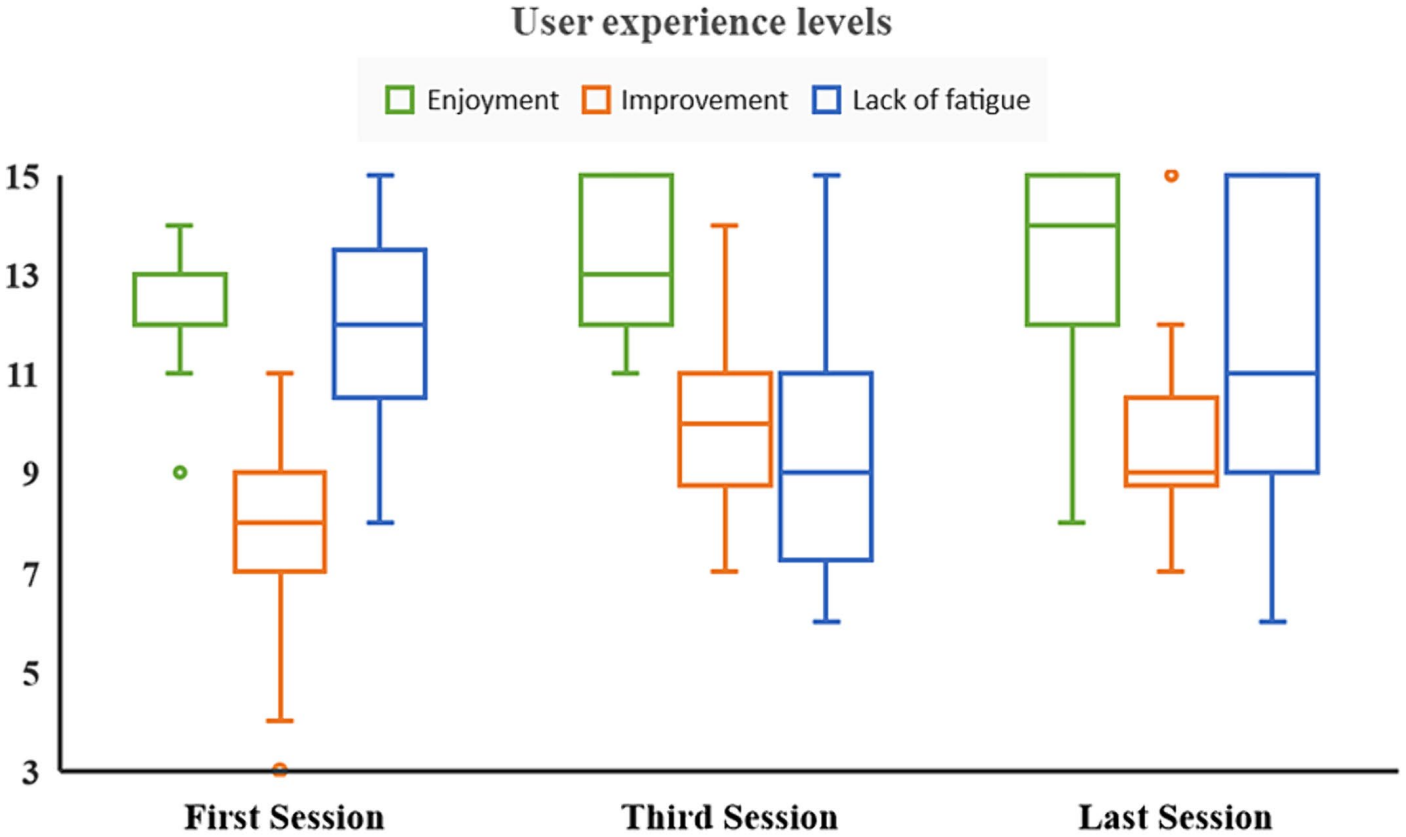
Boxplots of user experience levels during the immersive virtual reality intervention. Median scores of self-reported values of enjoyment, perceived improvement and lack of fatigue.

Friedman tests were conducted to examine differences in user experience levels, including enjoyment, perceived improvement, and fatigue, following the first, third and final sessions of the IVR multimodal intervention (Figure 4). The results indicated a statistically significant main effect of time (*χ*^2^(2) = 6.609, *p* = 0.04). Post-hoc analyses, reporting median (*Mdn*) and interquartile range (*IQR*) values, revealed a gradual increase in enjoyment levels from the first (*Mdn* = 12, *IQR* = 12.00, 13.55) to the third (*Mdn* = 13, *IQR* = 12.00, 15.00) and then to the final session (*Mdn* = 14, *IQR* = 12.00, 15.00), with consistently very high satisfaction ratings throughout the sessions. However, this progressive increase did not reach statistical significance when the assessment times were compared individually (i.e., differences between the first and third and final sessions, *p* > 0.05). Friedman tests were also conducted to examine differences in perceived improvement and fatigue levels across sessions. Perceived improvement scores remained high throughout the intervention, from the first (*Mdn* = 8, *IQR* = 7, 9.50) to the third (*Mdn* = 10, *IQR* = 8.25, 11.00) and then to the final session (*Mdn* = 9, *IQR* = 8.25, 11.50), but these differences were not statistically significant (*χ*^2^(2) = 3.000, *p* = 0.22). In the case of rest-fatigue levels, participants reported a slightly increasing pattern of fatigue levels from the first (*Mdn* = 12, *IQR* = 10.50, 13.50) to the third (*Mdn* = 9, *IQR* = 07.25, 11.00) and then to the final session (*Mdn* = 11, *IQR* = 9.00, 15.00), but these differences were not statistically significant (*χ*^2^(2) = 5.545, *p* = 0.062).

### 3.3 Generalizability of the results

Finally, since significant improvements were observed in some cognitive measures (e.g., global cognition, simple attention, processing speed, memory, and executive function) and mental health (e.g., depressive symptoms), we assessed whether these improvements could have been associated with a greater generalizability of the results to daily life situations and activities. We subtracted the pre-post assessment differences in the transformed, or scale, scores in the MoCA, Stroop Colors, TMT-A, RAVLT Sum, RAVLT immediate recall, and RAVLT delayed recall measures, to compute a mean difference score for each of the measures in which the experimental group reported significant improvements. Post-hoc Pearson’s product–moment correlations were run. There was only a statistically significant, moderately positive, correlation between depressive symptoms (PHQ-9 diff) and the generalization of the results (*r*(98) = 0.608, *p* = 0.03). However, there were moderate-to-strong significant positive correlations between most of the cognitive measures and depressive symptoms (Table 3).

**Table 3:** Pearson correlations between variables in the experimental group.

	1	2	3	4	5	6	7	8
MoCA diff.								
TMT-A diff.	0.454^*^							
Stroop C diff.	0.329	0.350						
RAVLT Sum diff.	0.459^*^	0.214	0.636^**^					
RAVLT IR diff.	0.469^*^	0.290	0.598^**^	0.842^**^				
RAVLT DR diff.	0.620^**^	0.387	0.567^**^	0.909^**^	0.822^**^			
PHQ-9 diff.	0.453^*^	0.192	0.393	0.716^**^	0.582^**^	0.658^**^		
Generalization	0.206	0.133	0.376	0.346	0.087	0.308	0.608^*^	

Pre-post computed differences (diff.) in cognition and mental health variables. Asterisks denote significant pre-post changes (*p < 0.05, **p < 0.01).

## 4 Discussion

To our knowledge, this innovative study represents the first attempt to assess the preliminary efficacy of a multimodal, group-based, IVR intervention - including cognitive training, physical exercise, and MBI - compared to a usual care intervention (control group). The primary objective of this study was to assess changes in several cognitive domains and mental health symptoms in patients with PCC, following a multimodal IVR intervention, and comparing this with a usual care intervention. Our research marks a promising advance in the treatment of PCC and paves the way for the ongoing development of IVR-based multimodal training interventions.

After the intervention, we observed significant improvements in certain cognitive functions, such as global cognition, processing speed and episodic memory, among participants in the experimental group when they were compared with the control group. These improvements were consistent with previous research using traditional paper-based and digital neuropsychological training methods involving patients with PCC (García-Molina et al., 2021; Rabaiotti et al., 2023). Like previous digital cognitive training interventions (García-Molina et al., 2021), our group-based IVR approach significantly improved episodic memory and processing speed and reduced depressive symptoms in PCC patients. Similarly, we found significant improvements in global cognitive function, as indicated by the MoCA; this was consistent with findings from a recent study that combined physical and cognitive training (Rabaiotti et al., 2023). Our findings further support the potential of cognitive training techniques based on neuroplasticity principles (Park and Bischof, 2013) to reverse cognitive impairments in PCC. However, our study also included a control group (i.e., usual care intervention). This potentially strengthened our findings compared to studies that used a single-group design (García-Molina et al., 2021; Rabaiotti et al., 2023). Beyond traditional neuropsychological rehabilitation methods, the use of IVR technology in cognitive training offers significant advantages over paper-based methods by increasing patient engagement and adherence through the delivery of dynamic, personalized, cognitive training programs—which are particularly beneficial for individuals with cognitive impairments and/or neurocognitive disorders (Coyle et al., 2015; Moreno et al., 2019). In addition, digital technologies enable real-time data collection and analysis to guide the development of personalized, dynamically adaptable, interventions (Mouton and Cloes, 2013). Some systematic reviews have highlighted the potential of these digital technologies to create not only more engaging interventions, but also potentially more effective cognitive training paradigms (Moreno et al., 2019; Ahmadi Marzaleh et al., 2022). For example, VR has the ability to simulate authentic social and environmental conditions, potentially enhancing the effectiveness of cognitive rehabilitation interventions (Ahmadi Marzaleh et al., 2022). By generating immersive and compelling scenarios, VR may induce a broader activation of neural networks that mirror those engaged during real-world experiences (Parsons et al., 2017). In addition, the controlled environment inherent in VR allows for the systematic control of cognitive challenges and the real-time observation of cognitive performance, promoting a more dynamic, individualized approach to cognitive training (Parsons et al., 2017).

This study also sought to investigate the effects of the multimodal IVR intervention on mental health and fatigue levels in participants with PCC. In addition to cognitive improvements, our intervention also produced significant improvements in depressive symptomatology among participants in the experimental group. The reported improvement in depressive symptoms may have been attributable to the combined effects of physical and cognitive training. This integrative approach has been highlighted for its efficacy in previous studies involving other populations to improve cognition and depressive symptoms (Zhu et al., 2016; Karssemeijer et al., 2017). Our study provides further support for these findings. We also found significant associations between postintervention gains in various cognitive domains and lower depressive symptoms, as well as a significant association between a more extensive generalization of cognitive gains in daily life and lower depressive symptoms. These findings highlight the significant influence of cognitive enhancement as a potential approach for reducing depressive symptoms (Simons et al., 2009; Pan et al., 2019) and thereby reinforcing the reciprocal, bidirectional relationship between cognition and depressive symptoms (Csajbók et al., 2023). Although our intervention showed significant efficacy in reducing depressive symptoms, it did not substantially affect anxiety symptoms in any group. The differential effect on depressive versus anxiety symptoms may be explained by the specific characteristics of our multimodal intervention. Although MBIs are typically considered effective in reducing anxiety, it appears that we may need to extend the duration of each session beyond the current 10 min to significantly reduce anxiety symptoms. Alternatively, it may be beneficial to supplement our approach with additional interventions. These could be designed to temper the heightened sensitivity of participants to perceived threats or to address their tendency to worry about the future (Khawam et al., 2020).

During the IVR sessions in our study, the fatigue levels reported by patients did not fluctuate significantly over the course of the intervention. Similarly, there were no significant differences between groups in terms of self-reported fatigue levels before and after the intervention. Although our study did not demonstrate a significant reduction in fatigue levels, it is noteworthy that the intensive weekly cognitive, physical and MBI training did not exacerbate fatigue any more than routine standard care. These results support the tolerability of our multimodal IVR intervention for adults with PCC. Our findings were consistent with previous systematic reviews and meta-analyses that have highlighted the usefulness of IVR in reducing the subjective impact of fatigue in clinical populations prone to high levels of fatigue, such as patients with fibromyalgia (Cortés-Pérez et al., 2021b) and multiple sclerosis (Cortés-Pérez et al., 2021a). IVR-based interventions, with their tailor-made therapeutic experiences, which actively engage patients in stimulating tasks, have the potential to distract patients from their feelings of fatigue and to enable them to focus more, and better, on the intervention (Cortés-Pérez et al., 2021b); this may consequently improve their tolerance to such interventions. Assessing tolerability is a critical factor when evaluating the feasibility of these technologically advanced interventions (Ahmadi Marzaleh et al., 2022), especially considering that fatigue is one of the primary symptoms experienced by many PCC patients (Davis et al., 2021). An intervention that does not exacerbate fatigue may therefore be particularly beneficial for this population.

When assessing user experience, our results were similarly generally positive, with participants reporting high to very high levels of enjoyment and perceived improvement with our multimodal intervention. This contrasts with many traditional cognitive training approaches, which have often been perceived as being boring or unengaging, and have required additional motivational features (Mohammed et al., 2017). When comparing our results with other VR studies that adopted a similar multimodal approach and involved participants with COVID-19 and PCC, we found that our intervention appeared to perform well. In line with our findings, these studies also reported high levels of usability and acceptability among their participants (Kolbe et al., 2021; Groenveld et al., 2022). However, it is important to highlight some of the differences between the studies in terms of the type of IVR technology used. While there is consensus about the ability of IVR technology to improve therapeutic outcomes for adults with a wide range of cognitive decline and mental health problems (Ahmadi Marzaleh et al., 2022), specific applications of this technology may vary. While providing an immersive and interactive experience, the use of individual, head-mounted displays may pose challenges for certain individuals. For example, older adults with PCC may lack the experience, skills and/or social support to effectively engage with this type of technology, potentially leading to feelings of exclusion rather than engagement (Czaja, 2016). Our study mitigated this problem by taking advantage of recent technological advances and utilizing a CAVE-based VR setup (i.e., an MK360 device; Linares-Chamorro et al., 2022). This solution, which integrates sophisticated hardware and software and includes an immersive content platform, enables the creation of multisensory experiences in real-world spaces, eliminating the need for individual VR head-mounted displays. This provides a unique opportunity to make this technology more accessible to patients with physical, cognitive and/or emotional limitations (Seifert et al., 2019). In addition, the use of a group setting offers significant advantages over individual VR sessions, including: social identification, motivation, behavioral adaptation, cohesion, trust and constructive feedback. This enhances interpersonal relationships and collective performance (Borek and Abraham, 2018). This innovative strategy may therefore optimize the implementation of VR technology in PCC rehabilitation.

### 4.1 Limitations

Despite these positive results, we found a lack of relationship between cognitive gains and the generalization of results to everyday situations. These findings call for additional research to better understand how cognitive gains can be effectively translated into everyday benefits for adults with PCC. Previous research using comparable digital, physical-cognitive training paradigms (e.g., exergames) has emphasized the need for realistic simulations involving natural and spontaneous tasks that reflect everyday contexts and situations, as this may facilitate transfers to everyday activities (Temprado, 2021; Perrot and Maillot, 2023). Our multimodal IVR intervention exposed participants to specific everyday-life scenarios, such as walking in parks. However, it may be beneficial to expand the range of environments to include a wider variety of emotionally relevant daily activities, and also to tailor our interventions in order to better reflect real-life problem-solving tasks and/or strategies that promote the application of learned skills to daily activities.

Another important limitation of this study was the use of a quasi-experimental design. The decision to assign participants sequentially, rather than randomly, was based on practical considerations and resource constraints. In this pilot study, our primary focus was on developing and testing the feasibility of our multimodal IVR program with patients exhibiting post-COVID-19 conditions. The randomization of the participants and blinding of the evaluators would have required significant additional resources and personnel that were beyond our budget. Instead, our limited resources were focused on refining and validating the IVR technology to ensure the effective delivery of the intervention. This design does, however, have a number of inherent limitations. One of these involves establishing a causal relationship between an intervention and an outcome and the potential for selection bias and confounding variables (Harris et al., 2006). Future research should aim to use a randomized controlled trial design and blinding evaluators. It should thereby be possible to replicate and further strengthen the observed improvements in cognitive and mental health. The relatively small sample size placed another limitation on the generalizability of our findings. Future studies should seek to include larger, more diverse, groups of participants in order to ensure the broader applicability of the findings. In addition, the control group condition was not fully aligned for comparison with the multimodal IVR intervention. Future work should replicate this study, using a control group that could provide identical cognitive training, physical exercise, and mindfulness-based tasks but without the use of an IVR device. This may help to further strengthen our initial findings.

In addition, we found some notable, albeit mostly nonsignificant, differences between groups in terms of their pre-assessment scores. Overall, the experimental group reported higher clinical symptomatology than the control group. Future research should ensure the homogeneity of baseline characteristics through a randomized experimental design to minimize potential confounding effects. Given the pilot nature of this study, which was primarily designed to assess feasibility and provide preliminary estimates of intervention effects, an *a priori* power analysis was not performed to calculate sample size. In addition, this study did not account for other potential confounding factors, such as the monitoring of the medication status of the participants during the study, the onset and progression of the COVID-19 disorder, the presence of other clinical comorbid disorders, or the prior technology background and technology use skills of the participants. These uncontrolled variables could have influenced the results and suggest areas that will require more rigorous control in future research.

## 5 Conclusion

Despite the inherent limitations of our study, its findings mark a pioneering step toward improving cognition and mental health outcomes in PCC through the innovative use of technology and multimodal methods, highlighting the importance of patient engagement in these cognitive training paradigms. These findings also provide important insights for the healthcare system, particularly relevant in the ongoing COVID-19 crisis, which has exponentially increased the need for remote and digital healthcare solutions (Torous et al., 2020). Our multimodal intervention provides an innovative way to address this need and highlights the critical role of cognitive and mental health interventions for adults with PCC, a vulnerable population due to the detrimental effects of the pandemic. This initial study should be accompanied by larger clinical trials aimed at further exploring and refining these interventions, thus contributing to a more inclusive, supportive and effective healthcare system amidst the ongoing COVID-19 global health crisis.

## Data availability statement

The raw data supporting the conclusions of this article will be made available by the authors, without undue reservation.

## Ethics statement

The studies involving humans were approved by the Ethical Review Board of the Terrassa Health Consortium—02-22-107-029. The studies were conducted in accordance with the local legislation and institutional requirements. The participants provided their written informed consent to participate in this study. Written informed consent was obtained from the individual(s) for the publication of any identifiable images or data included in this article.

## Author contributions

NC: Writing – review & editing, Writing – original draft, Project administration, Methodology, Investigation, Data curation, Conceptualization. JG-H: Writing – review & editing, Writing – original draft, Investigation, Data curation, Conceptualization. MA: Writing – review & editing, Writing – original draft, Visualization, Supervision, Methodology, Formal analysis, Data curation, Conceptualization. TM: Writing – review & editing, Writing – original draft, Supervision, Methodology, Formal analysis. DR: Writing – review & editing, Writing – original draft, Methodology, Formal analysis. BP-G: Writing – review & editing, Writing – original draft, Visualization, Supervision, Methodology, Investigation, Formal analysis, Data curation. MG: Writing – review & editing, Writing – original draft, Validation, Supervision, Resources, Project administration, Methodology, Investigation, Funding acquisition, Conceptualization.
